# Thyroid fine-needle aspiration biopsy positively correlates with increased diagnosis of thyroid cancer in South Korean patients

**DOI:** 10.1186/s12885-017-3104-0

**Published:** 2017-02-07

**Authors:** Yoon Jae Cho, Do Young Kim, Eun-Cheol Park, Kyu-Tae Han

**Affiliations:** 1 Premedical Courses, Yonsei University College of Medicine, Seoul, Republic of Korea; 2 Institute of Health Services Research, Yonsei University, Seoul, Republic of Korea; 3 Department of Preventive Medicine, Yonsei University College of Medicine, Seoul, Republic of Korea; 4 Department of Public Health, Graduate School, Yonsei University, Seoul, Republic of Korea

**Keywords:** Thyroid cancer, Biopsy, Fine-needle, Health Services Accessibility, Overdiagnosis

## Abstract

**Background:**

The incidence of thyroid cancer among South Koreans is more than 10-fold greater than its incidence in other countries, although its associated mortality rate is similar. Amidst concerns regarding the over-diagnosis of thyroid cancer related to gradually expanded medical testing in South Korea, we hypothesized that the number of thyroid fine-needle aspiration biopsies has led to increased diagnosis of thyroid cancer.

**Methods:**

We used data from the National Health Insurance Service National Sample Cohort 2003–2013, which included all medical claims filed for the 1,122,456 people in a nationally representative sample. We performed a Poisson regression analysis using generalized estimating equation to investigate the relationship between the number of thyroid fine-needle aspiration biopsies and the newly diagnosed cases of thyroid cancer.

**Results:**

The study included 60 annual patients per 100,000 individuals out of 11,024,548 person-years. The number of biopsies per 100,000 patients positively correlated with increased incidence of thyroid cancer diagnosis (per 100 biopsy cases: RR = 1.108; 95% CI: 1.090–1.126; *P* < 0.0001). Such relationships were greater in males, patients with a higher socioeconomic status, and patients from regions with relatively less accessibility to biopsies.

**Conclusion:**

Our findings suggest that a higher number of thyroid fine-needle aspiration biopsies per 100,000 individuals in a specific Si-Gun-Gu is positively associated with excessively increased diagnosis of thyroid cancer. Regarding the continually increasing thyroid cancer incidence in South Korea, healthcare professionals and policy makers should consider proper guidelines for recognizing the role of thyroid fine-needle aspiration biopsies in the potential over-diagnosis of thyroid cancer.

**Electronic supplementary material:**

The online version of this article (doi:10.1186/s12885-017-3104-0) contains supplementary material, which is available to authorized users.

## Background

South Korea has experienced rapid modernization both socially and economically, leading to the improved health status of South Koreans but an increase of elderly individuals [1, 2]. As a result, the dominant disease patterns of South Koreans shifted from communicable diseases to non-communicable diseases [3], such as cancer [4, 5]. Many South Koreans now participate in preventive “health checkup” programs, which can positively affect cancer-related health outcomes. However, increased medical testing has led to an unexpected challenge: the “over-diagnosis” of asymptomatic cancers in South Korean individuals [6, 7].

Over-diagnosis occurs when a condition is diagnosed that would otherwise not produce symptoms or cause death [8] and has been tentatively observed with respect to thyroid cancer [9]. According to GLOBOCAN, the incidence of thyroid cancer in South Korea was more than 10-fold greater than in other countries, although its mortality rate is similar (incidence: 52.8 per 100,000 South Koreans, 4.0 per 100,000 people worldwide; mortality: 0.5 per 100,000 individuals) [10, 11]. In addition, the incidence has rapidly increased in South Korea (6.9 vs. 71.3 per 100,000 people in 2000 and 2013, respectively) in parallel with increased medical utilization during this time [12]. Therefore, many healthcare professionals have investigated the possible causes of such rapid increases. The increased incidence of small papillary thyroid cancer with an unchanged mortality rate [13, 14] suggests that it may result due to more frequent thyroid cancer screenings, improved diagnostic scrutiny, increased coverage of the National Health Insurance (NHI), more accessibility to ultrasonography, and certain environmental and genetic factors [6, 15].

In South Korea, cancer screenings, including thyroid biopsy, are often performed to confirm abnormal findings based on ultrasonography or other clinical indications [16]. According to the National Health Insurance Service (NHIS), the number of thyroid fine-needle aspiration biopsies increased in parallel to the increase in newly diagnosed cases of thyroid cancer [17]. Nevertheless, there are no alternatives for controlling such increases in fine-needle aspiration biopsy and thyroid cancer, and more detailed studies are required to establish effective alternatives for optimal management of thyroid cancer. We hypothesized that the increase in biopsies could significantly affect diagnosis of thyroid cancer, and possibly lead to overdiagnosis. The current study aims to identify an increase in the number of unnecessary thyroid fine-needle aspiration biopsies, and to determine whether it contributes to increasing diagnosis of thyroid cancer.

## Methods

### Study population

The data used in this study were obtained from the NHIS National Sample Cohort 2002–2013 released in 2014 and include a nationally representative random sample of 1,025,340 individuals, approximately 2.2% of the entire NHIS population in 2002. The data were compiled by the NHIS using a systematic sampling method to generate a representative sample of 46,605,433 Korean residents. The database includes all medical claims filed from January 2002 to December 2013. To investigate the relationship between the number of thyroid fine-needle aspiration biopsies in each geographic region and newly diagnosed cases of thyroid cancer, we excluded patients who were diagnosed with thyroid cancer (ICD-10: C73) before 2003. We then identified patients who underwent a thyroid biopsy (EDI code: C8591) and aggregated this number as a unit of 253 basic administrative districts (Si-Gun-Gu; city-county-ward) of South Korea. Data used in this study consisted of 11,024,548 person-years of 1,122,456 individuals during 2003–2013.

### Variables

Our outcome variable was the number of newly diagnosed cases of thyroid cancer during the study period, indicated by the first hospital visit during which thyroid cancer (ICD-10: C73) was the major diagnosis for each patient.

The primary independent variable was the number of fine-needle aspiration biopsies performed in each Si-Gun-Gu. We first identified whether patients received thyroid needle aspiration biopsies based on EDI Code and aggregated the number of biopsies as a unit of Si-Gun-Gu per each year. We then calculated the number of biopsies per 100,000 patients using the following formula:

$$ =\frac{{\displaystyle \sum } Thyroid\  fine\  needle\  aspiration\  biopsy\  in\  Si- Gu n- Gu}{The\  number\  of\  population\  in\  Si- Gu n- Gu} \times 100,000 $$

We also adjusted other independent variables when analyzing the association between the number of biopsies per 100,000 people and cases of newly diagnosed thyroid cancer. Other independent variables included sex, age, income, type of insurance coverage, study year, region, and the financial independence rate of the local government. Ages were categorized as ≤19, 20–29, 30–39, 40–49, 50–59, 60–69, 70–79, and ≥80 years to reflect differences in diagnosis of thyroid cancer [18]. The types of insurance coverage were categorized as medical aid, NHI employee insurance, or NHI self-employed insurance based on NHI criteria. Those with NHI employee insurance included workers and employers in all workplaces, public officials, private school employees, continuously insured persons, and daily paid workers at construction sites. Beneficiaries of NHI employee insurance included spouses, descendants, siblings, and parents. Individuals with NHI employee insurance paid approximately 7% of their average salary in contribution payments, though these rates usually changed annually. The NHI self-employed insurance category included people whose contribution amount was set based on their income, property, living standard, and rate of participation in economic activities. Medical aid beneficiaries were patients with an income below the government-defined poverty level or who had a disability and were provided with free in- and outpatient care via government funds. Therefore, the type of insurance coverage represented each patient’s socioeconomic status [2]. We included this variable in the study to consider potential differences in the accessibility of thyroid cancer screening according to socioeconomic status. The financial independence rate of the local government was an index of the finance utilization capacity of a local government with independent discretionary power, which was calculated as: (local taxes + non-tax revenue)/local government budgets × 100 [19].

### Statistical analysis

We first examined the frequencies and percentages of each categorical variable or the mean and standard deviation of each continuous variable at each patient’s baseline, respectively. We performed χ2 tests to analyze the distribution of person-years for each categorical variable by diagnosis of thyroid cancer and an analysis of variance (ANOVA) for each continuous variable by diagnosis during the study period. The tests were performed in all study subjects and patients who received thyroid biopsy, respectively. Finally, we performed Poisson regression analysis using generalized estimating equations (GEE) to investigate the relationship between the number of thyroid biopsies and cases of newly diagnosed thyroid cancer adjusting for sex, age, income, type of insurance coverage, study year, region, and financial independence rate of regional government. GEE models with link logit that included both patient- and region-level variables were analyzed, as data used in this study were hierarchically structured and had binary outcome variables. This model assumed proper distributions for each hospitalization case while taking into account the correlation among individuals within the Si-Gun-Gu. In this study, the correlation was an exchangeable correlation structure [20]. To identify whether thyroid biopsies were unnecessary for diagnosis of thyroid cancer, we also analyzed the relationship between the number of thyroid biopsies and newly diagnosed thyroid cancer cases only among patients who received thyroid biopsies. The goodness-of-fit for the GEE model was assessed using the quasi-likelihood under the independence criterion (QIC), whose lower value indicated the goodness-of-fit [21]. In addition, we performed sub-group analyses for Poisson regression analyses to compare differences in the association between the number of biopsies and cases of newly diagnosed thyroid cancer according to sex, income, the median number of thyroid fine-needle biopsies, and financial independence rate of the local government. All statistical analyses were performed using SAS version 9.4.

## Results

**Fig. 1:**
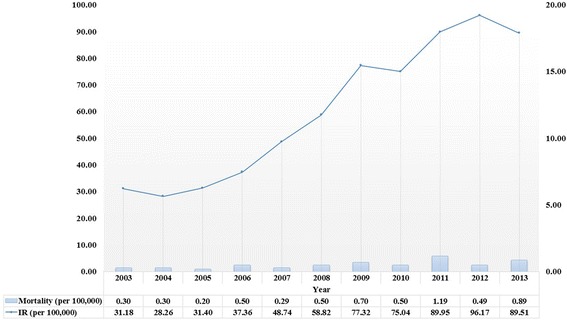
Trends of annual thyroid cancer incidence and mortality during 2003–2013

**Fig. 2:**
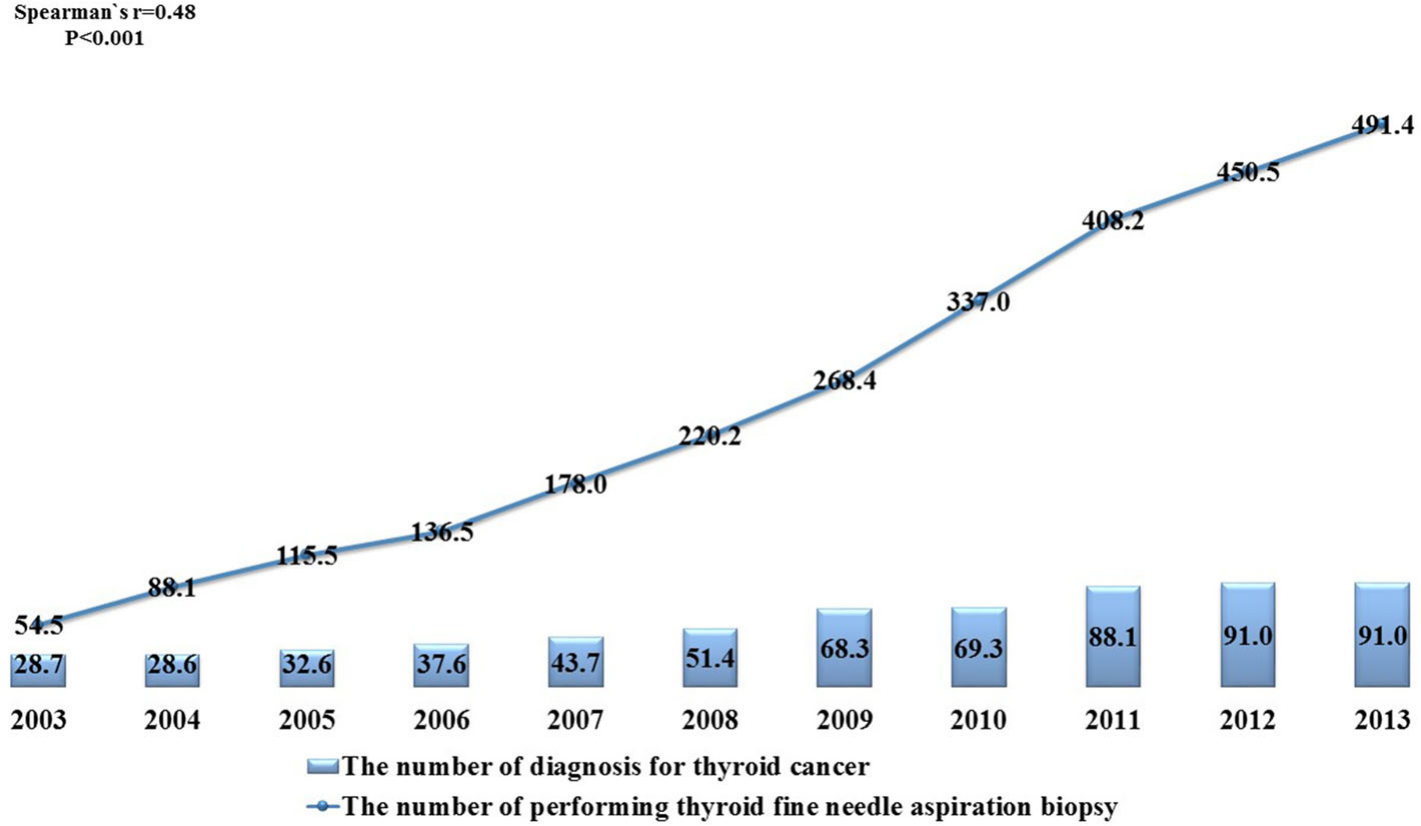
Correlation between thyroid fine-needle aspiration biopsy frequency and diagnosis of thyroid cancer during 2003–2013. *Each indicator was calculated as the number of thyroid fine-needle aspiration biopsy or diagnosis of thyroid cancer per 100,000 individuals in Si-Gun-Gu

The data used in this study were compiled from 1,122,456 people at baseline and represented 11,024,548 person-years during the study period. Additional file 1 shows the patients’ general characteristics, including individual- and regional-level variables at baseline. The average follow-up period of each person included in this study was 9.82 person-years. The average number of thyroid fine-needle aspiration biopsies in each Si-Gun-Gu at baseline was 73.16 per 100,000 individuals. There were generally more individuals in the lower age group than in the older age groups. “NHI employed” was the most common type of insurance coverage. Figure 1 shows trends of the incidence and mortality of thyroid cancer during the study period. The incidence gradually increased, but the mortality rate remained relatively stable. Figure 2 shows the positive correlation between number of thyroid fine-needle aspiration biopsies and new diagnoses of thyroid cancer during the study period (Spearman correlation coefficient: 0.48, *P* < 0.001).

Table 1 shows the associations between new cases of thyroid cancer and each independent variable in this study. We observed a 0.6% incidence rate (*n* = 6619 diagnosed patients) among 11,024,548 person-years, and the average number of thyroid fine needle biopsies in Si-Gun-Gu was greater in patients diagnosed with thyroid cancer than in patients who were not diagnosed (Diagnosed mean: 348.2, SD: 225.0; Non-diagnosed mean: 253.3, SD: 207.9; *P* < 0.0001). In addition, socioeconomic status had a positive linear association with thyroid cancer diagnosis. By region, patients from Jeollanam-do were more frequently with thyroid cancer than patients from other regions. On the other hand, in regards to patients with thyroid biopsy, the average number of thyroid fine-needle biopsies performed in Si-Gun-Gu was lower in patients diagnosed with thyroid cancer compared to others.

**Table 1:** Distribution of person-years by diagnosis of thyroid cancer

Variables	Total patients					Patients with thyroid fine-needle aspiration biopsy				
	Diagnosed		None		*P*-value	Diagnosed		None		*P*-value
	N/Mean	%/SD	N/Mean	%/SD		N/Mean	%/SD	N/Mean	%/SD	
Regional variables										
Number of thyroid fine-needle aspiration biopsies in Si-Gun-Gu (per 100,000 people)	348.2	225.0	253.3	207.9	<0.0001^a^	348.2	225.0	425.9	236.8	<.0001^a^
Financial independence rate of local government (%)	62.2	23.3	62.4	23.6	0.5344^a^	62.2	23.3	59.4	22.9	<.0001^a^
Individual variables										
Sex										
Male	1140	0.02	5,514,264	99.98	<0.0001^b^	1140	22.04	4033	77.96	0.1968^b^
Female	5479	0.10	5,503,665	99.90		5479	21.23	20,327	78.77	
Age (years)										
0–19	30	0.00	2,622,533	100.00	<0.0001^b^	30	21.58	109	78.42	<0.0001^b^
20–29	339	0.02	1,580,567	99.98		339	26.26	952	73.74	
30–39	1210	0.07	1,866,884	99.94		1210	26.86	3295	73.14	
40–49	1972	0.10	1,909,614	99.90		1972	23.69	6352	76.31	
50–59	1819	0.13	1,392,449	99.87		1819	19.48	7519	80.52	
60–69	876	0.10	894,668	99.90		876	16.65	4386	83.35	
70–79	320	0.06	543,227	99.94		320	16.82	1582	83.18	
80+	53	0.03	207,987	99.98		53	24.31	165	75.69	
Type of insurance coverage										
Medical Aid	144	0.04	383,596	99.96	<0.0001^b^	144	34.37	275	65.63	<0.0001^b^
NHI (self-employed)	2142	0.05	4,060,517	99.95		2142	21.69	7734	78.31	
NHI (employed)	4333	0.07	6,573,816	99.93		4333	20.95	16,351	79.05	
Income (percentiles)										
0–29%	873	0.05	1,784,071	99.95	<0.0001^b^	873	20.45	3395	79.55	0.2938^b^
30–59%	1310	0.05	2,612,723	99.95		1310	21.49	4785	78.51	
60%+	4436	0.07	6,621,135	99.93		4436	21.52	16,180	78.48	
Year										
2003	317	0.03	1,016,565	99.97	<0.0001^b^	317	40.80	460	59.20	<0.0001^b^
2004	287	0.03	1,015,716	99.97		287	26.70	788	73.30	
2005	319	0.03	1,015,929	99.97		319	23.74	1025	76.26	
2006	374	0.04	1,001,078	99.96		374	23.29	1232	76.71	
2007	497	0.05	1,019,688	99.95		497	22.42	1720	77.58	
2008	588	0.06	999,651	99.94		588	22.13	2069	77.87	
2009	771	0.08	997,219	99.92		771	24.12	2425	75.88	
2010	751	0.08	1,000,753	99.93		751	19.37	3126	80.63	
2011	884	0.09	983,859	99.91		884	20.49	3430	79.51	
2012	941	0.10	983,065	99.90		941	19.37	3918	80.63	
2013	890	0.09	984,406	99.91		890	17.60	4167	82.40	
Region (distance from Seoul)										
Gangwon-do (100.6 km)	115	0.04	321,209	99.96	<0.0001^b^	115	20.25	453	79.75	<0.0001^b^
Gyeonggi-do (40.0 km)	1445	0.06	2,528,926	99.94		1445	21.80	5182	78.20	
Gyeongsangnam-do (366.4 km)	335	0.05	653,458	99.95		335	20.68	1285	79.32	
Gyeongsangbuk-do (225.5 km)	303	0.05	595,692	99.95		303	22.35	1053	77.65	
Gwangju (295.3 km)	272	0.08	324,124	99.92		272	14.48	1607	85.52	
Daegu (288.3 km)	432	0.08	561,837	99.92		432	17.63	2019	82.37	
Daejeon (160.9 km)	267	0.08	334,572	99.92		267	28.13	682	71.87	
Busan (394.2 km)	440	0.05	807,140	99.95		440	19.32	1837	80.68	
Seoul	1504	0.07	2,282,278	99.93		1504	24.78	4566	75.22	
Ulsan (395.7 km)	184	0.07	259,663	99.93		184	24.02	582	75.98	
Incheon (37.7 km)	265	0.04	606,840	99.96		265	23.47	864	76.53	
Jeollanam-do (346.3 km)	382	0.09	429,219	99.91		382	17.54	1796	82.46	
Jeollabuk-do (216.9 km)	244	0.06	413,454	99.94		244	18.26	1092	81.74	
Jeju-do (541.6 km)	69	0.06	123,336	99.94		69	23.31	227	76.69	
Chungcheongnam-do (129.9 km)	226	0.05	437,945	99.95		226	24.73	688	75.27	
Chungcheongbuk-do (137.1 km)	136	0.04	338,236	99.96		136	24.16	427	75.84	
Total	6619	0.06	11,017,929	99.98		6619	21.37	24,360	78.63	

^a^The results of analysis of variance (ANOVA) for each continuous variable to compare mean and standard deviation by diagnosis during study period

^b^The results of χ2 tests to analyze frequencies of person-years for each categorical variable by diagnosis of thyroid cancer

Table 2 shows the results of GEE Poisson regression analyses for the entire population and for patients with thyroid needle biopsy, respectively. In the whole population, the number of biopsies per 100,000 individuals was positively associated with diagnosis of thyroid cancer (per 100 cases: RR = 1.108, 95% CI: 1.090-1.126; *P* < 0.0001). The financial independence rate of the local government was also positively associated with increased diagnosis of thyroid cancer but it was not statistically significant. Diagnosed cases of thyroid cancer in females were 5-fold greater than males, and patients 40–59 years of age were more often diagnosed than patients of other age groups. Patients of higher socioeconomic status showed a greater incidence of thyroid cancer diagnosis. In addition, the risk in the diagnosis of thyroid cancer was gradually increased by the year. In patients who received thyroid biopsy, in contrast to results from the entire population, the regional number of thyroid fine-needle aspiration biopsy was inversely associated with the diagnosis of thyroid cancer (per 100 cases: RR = 0.973, 95% CI: 0.952-0.995; *P* = 0.0143). In particular, patients under 40 years of age were more often diagnosed than patients in other age groups.

**Table 2:** Poisson regression analysis results for diagnosis for thyroid cancer

Variables	Total patients				Patients with thyroid fine-needle aspiration biopsy			
	RR^a^	95% CI		*P*-value	RR^a^	95% CI		*P*-value
Regional variables								
Number of thyroid fine-needle aspiration biopsy in Si-Gun-Gu (per 100,000 people; per 100 increase)	1.108	1.090	1.126	<0.0001	0.973	0.952	0.995	0.0143
Financial independence rate of local government (per 10%)	1.037	0.955	1.125	0.3849	1.003	0.992	1.015	0.5599
Individual variables								
Sex								
Male	0.206	0.194	0.220	<0.0001	1.075	0.990	1.168	0.0852
Female	1.000	-	-	-	1.000	-	-	-
Age (years)								
0–19	0.059	0.038	0.093	<0.0001	1.359	0.703	2.627	0.3613
20–29	1.155	0.864	1.543	0.3313	1.700	1.065	2.713	0.0263
30–39	3.334	2.532	4.391	<0.0001	1.796	1.142	2.824	0.0113
40–49	5.389	4.100	7.082	<0.0001	1.519	0.968	2.385	0.0689
50–59	6.430	4.892	8.450	<0.0001	1.219	0.776	1.913	0.3897
60–69	4.721	3.578	6.230	<0.0001	0.921	0.584	1.453	0.7245
70–79	2.542	1.901	3.398	<0.0001	0.858	0.535	1.376	0.5248
80+	1.000	-	-	-	1.000	-	-	-
Type of insurance coverage								
Medical Aid	0.768	0.642	0.919	0.0040	1.466	1.101	1.952	0.0087
NHI (self-employed)	0.785	0.745	0.827	<0.0001	1.014	0.947	1.085	0.6984
NHI (employed)	1.000	-	-	-	1.000	-	-	-
Income (percentiles)								
0–29%	0.640	0.591	0.692	<0.0001	0.879	0.794	0.974	0.0135
30–59%	0.725	0.682	0.771	<0.0001	0.965	0.892	1.045	0.3839
60%+	1.000	-	-	-	1.000	-	-	-
Year								
2003	1.000	-	-	-	1.000	-	-	-
2004	0.853	0.727	1.001	0.0517	0.784	0.597	1.029	0.0795
2005	0.907	0.776	1.060	0.2187	0.770	0.593	1.000	0.0500
2006	1.029	0.885	1.197	0.7092	0.878	0.686	1.125	0.3051
2007	1.258	1.086	1.456	0.0022	0.924	0.727	1.174	0.5179
2008	1.438	1.243	1.664	<0.0001	0.927	0.730	1.178	0.5372
2009	1.765	1.530	2.035	<0.0001	1.023	0.807	1.297	0.8505
2010	1.583	1.361	1.842	<0.0001	0.870	0.680	1.112	0.2646
2011	1.757	1.509	2.045	<0.0001	1.026	0.804	1.309	0.8379
2012	1.725	1.477	2.015	<0.0001	1.041	0.813	1.332	0.7510
2013	1.579	1.345	1.854	<0.0001	1.067	0.830	1.371	0.6152
Region (distance from Seoul)								
Gangwon-do (100.6 km)	1.001	0.662	1.515	0.9960	0.999	0.557	1.789	0.9959
Gyeonggi-do (40.0 km)	1.192	1.042	1.364	0.0107	0.955	0.792	1.151	0.6277
Gyeongsangnam-do (366.4 km)	1.234	0.925	1.647	0.1526	1.130	0.760	1.680	0.5457
Gyeongsangbuk-do (225.5 km)	1.293	0.882	1.897	0.1879	1.322	0.777	2.249	0.3028
Gwangju (295.3 km)	1.435	1.124	1.832	0.0037	0.882	0.634	1.226	0.4537
Daegu (288.3 km)	1.325	1.099	1.598	0.0032	1.106	0.850	1.437	0.4539
Daejeon (160.9 km)	1.735	1.451	2.075	<0.0001	1.445	1.130	1.848	0.0033
Busan (394.2 km)	1.048	0.883	1.244	0.5927	1.238	0.974	1.574	0.0810
Seoul	1.238	1.003	1.528	0.0468	1.038	0.776	1.388	0.7999
Ulsan (395.7 km)	1.354	1.121	1.636	0.0017	1.370	1.074	1.746	0.0111
Incheon (37.7 km)	1.000	-	-	-				
Jeollanam-do (346.3 km)	1.774	1.150	2.737	0.0095	1.387	0.751	2.562	0.2954
Jeollabuk-do (216.9 km)	1.398	0.924	2.113	0.1125	1.263	0.710	2.245	0.4275
Jeju-do (541.6 km)	1.535	0.996	2.366	0.0522	1.436	0.794	2.598	0.2315
Chungcheongnam-do (129.9 km)	1.383	0.982	1.946	0.0633	1.239	0.772	1.990	0.3746
Chungcheongbuk-do (137.1 km)	1.130	0.780	1.636	0.5188	0.960	0.564	1.634	0.8805
QIC	99756.63				24319.41			

^a^Relative risk for diagnosis of thyroid cancer, based on the results of Poisson regression analysis with GEE adjusted for individual- and regional-level characteristic to identify the relationship between regional thyroid fine-needle biopsy rates and diagnosis of thyroid cancer

**Fig. 3:**
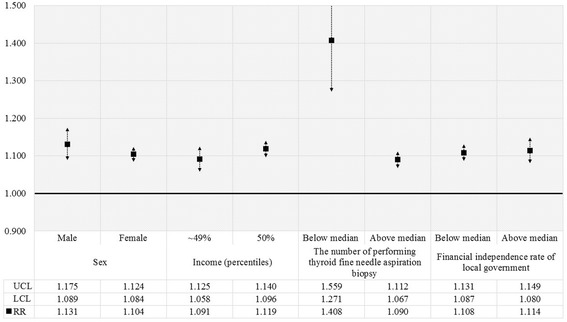
Results of subgroup analysis for all patients. *Results of subgroup analyses for the relationship between thyroid fine-needle aspiration biopsy and diagnosis of thyroid cancer among total patients according to sex, income, financial independence rate of the local government, and median number of thyroid fine-needle aspiration biopsies. Relative risk (RR) was calculated using Poisson regression analysis with GEE adjusted for individual- and regional-level characteristics. Results were considered statistically significant if each bar marked to SD did not reach the cutoff line of 1.00

**Fig. 4:**
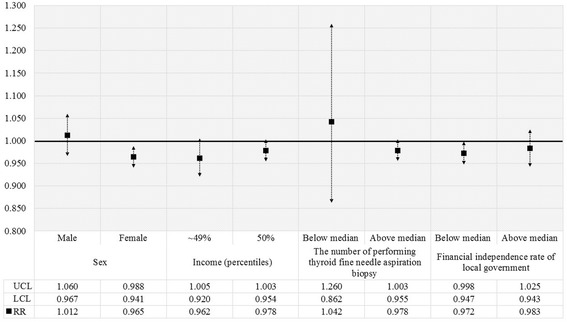
Results of subgroup analysis for patients who underwent thyroid fine-needle biopsy. * Results of subgroup analyses for the relationship between thyroid fine-needle aspiration biopsy and diagnosis of thyroid cancer only among patients who underwent thyroid fine-needle biopsy according to sex, income, financial independence rate of the local government, and median number of thyroid fine-needle aspiration biopsies. Relative risk (RR) was calculated by Poisson regression analysis with GEE adjusted for individual- and regional-level characteristics. Results were considered statistically significant if each bar marked to SD did not reach the cutoff line of 1.00

We also performed subgroup analyses to investigate positive associations in the number of biopsies with thyroid cancer diagnoses according to sex, income, median number of thyroid fine-needle biopsy, and financial independence rate of local government (Fig. 3). In the whole population, positive association was greater in males than females, in patients with incomes above the median financial independence rate, and in subjects from regions with lower biopsy frequencies than the median number. On the other hand, for patients who received thyroid biopsy, negative association was observed more in females as well as in patients with incomes below the median financial independence rate (Fig. 4).

## Discussion

The rapid improvement of health status in South Korean has created an “aging society” in which dominant health problems and issues have shifted to non-communicable diseases, such as cancer. Although many healthcare professionals have prompted positive outcomes through improved medical care [4], some concerns regarding the over-diagnosis of certain diseases, such as thyroid cancer, have arisen and have been validated in previous studies [7]. For example, previous studies suggest that increased access to ultrasonography in South Korea could contribute to increased cases of thyroid cancer [6]. However, questions remain regarding the environmental and genetic factors that may prompt the over-diagnosis of thyroid cancer.

We focused on the relationship between thyroid biopsies and newly diagnosed cases of thyroid cancer using nationwide sampling data and identified a positive correlation. Our results corroborate those of previous studies regarding the role of certain diagnostic tests, such as ultrasonography, in increased thyroid cancer diagnoses [22]. However, thyroid ultrasonography is not included under NHI coverage. Although increased diagnoses could be a natural result of more frequent screening procedures [23, 24], the increased diagnosis of small papillary thyroid cancer and other non-fatal thyroid cancers should still be investigated in South Korea because of a concomitant increase in preventive medical procedures and changes in thyroid cancer biopsy criteria [25]. However, regarding the more thyroid fine-needle aspiration biopsy were inversely associated with diagnosis of thyroid cancer among patients who received these biopsy, there might be excessive medical screening such as biopsy compared to actual diagnosis. Thus, there are needed to optimal control for guideline related to implementation of biopsy even if there were some controversies related to that.

The results of our sub-group analyses support our hypothesis, as the positive association between biopsy frequency and new cases of thyroid cancer was greater in patients with higher socioeconomic status, suggesting that greater accessibility to certain screening tests directly influences the frequency of cancer diagnosis [15]. Also, in the results for sub-group analysis by sex, regarding the incidence of thyroid cancer in females was higher than males based on previous studies, the increasing thyroid biopsy might cause to unnecessary increasing the diagnosis of thyroid cancer based on the greater positive correlations with diagnosis of thyroid cancer in males [18]. Meanwhile, subgroup analysis results for patients with biopsy showed that female patients and patients from low economic status areas had less diagnosis of thyroid cancer, in contrast to increase in regional biopsy. The results also suggested that unnecessary biopsy may be provided for patients at high risk of thyroid cancer or those with less health information.

Our study’s strengths include the use of national sampling cohort data to identify the relationship between the number of thyroid fine-needle aspiration biopsies and increased diagnoses of thyroid cancer. Therefore, our results are especially helpful for establishing evidence-based policies for managing thyroid cancer. Second, to our knowledge, this study is the first published attempt to investigate the impact of thyroid fine-needle aspiration biopsy frequency in individual geographic regions with respect to new cases of thyroid cancer. Previous studies focused on increased thyroid cancer incidence and changes in cancer type according to year or due to increased ultrasonography availability [6]. Thus, our findings could suggest another factor that contributes to more new thyroid cancer cases in South Korea. Third, our study analyzed the relationship between thyroid biopsies and thyroid cancer diagnoses adjusting for other covariates, such as socioeconomic status. Although other studies have linked increased thyroid cancer with differences in socioeconomic-related healthcare accessibility [15], we further analyzed the effects of income level, type of insurance coverage, and financial independence rate of the local government in this study.

Our study also has limitations. First, previous studies considered the types and size of thyroid cancer and accessibility to ultrasonography as important factors in over-diagnosis. However, we were unable to include these variables because these data were not available in the NHI database. Second, to identify overdiagnosis of cancer, information such as types and stages of thyroid cancer would be important. However, due to limited data, we could not identify such factors. Third, patients’ participation in the health checkup program could contribute to over-diagnosis of thyroid cancer, but we could not identify which patients were diagnosed with thyroid cancer through this program due to data limitations, even though incidence rates increased by year. Finally, income level data were only collected as units of 10th percentiles. Because income level appears to be a significant factor in thyroid cancer over-diagnosis, more specific income information for each patient could strengthen are study.

Despite these limitations, our findings suggest that increased numbers of thyroid fine- needle aspiration biopsies per 100,000 patients by geographic region could contribute to increased diagnoses of thyroid cancer in South Korea. Specifically, such relationships were more significant in males, patients with higher socioeconomic status, and in patients from regions with relatively less accessibility to biopsies. In addition, we also found that some excessive biopsies might be provided for people without increasing diagnosis among patients who received biopsies. The increased incidence of new thyroid cancer cases by year necessitates guidelines for optimal control and diagnosis of thyroid cancer and should prompt healthcare professionals and policy makers to consider the factors that contribute to excessive diagnosis of asymptomatic and nonfatal thyroid cancer.

## Conclusion

Our findings suggest that a higher number of thyroid fine-needle aspiration biopsies in each Si-Gun-Gu is positively associated with increased diagnoses of thyroid cancer in South Korean patients. We recommend that healthcare professionals and policy makers implement alternate preventive strategies to thyroid fine-needle aspiration biopsies during health checkup program visits.

## Additional file

Additional file 1: General characteristics of study population at baseline Provides a baseline characteristics of study population. (DOCX 16 kb)

## Abbreviation

ANOVA: Analysis of variance
CI: Confidence interval
EDI: Electronic data interchange
GEE: Generalized estimated equations
ICD: International classification of diseases
NHI: National Health Insurance
NHIS: National Health Insurance Service
QIC: Quasi-likelihood under the independence criterion
RR: Relative risk
SD: Standard deviation
