# Supplementary material for: Thyroid fine-needle aspiration biopsy positively correlates with increased diagnosis of thyroid cancer in South Korean patients
Source: BMC Cancer. 2017 Feb 7;17:114. doi: 10.1186/s12885-017-3104-0 (PMC5296957; doi:10.1186/s12885-017-3104-0)
Supplement: Additional file 1: — General characteristics of study population at baseline Provides a baseline characteristics of study population. (DOCX 16 kb) [file 12885_2017_3104_MOESM1_ESM.docx]

| **Additional file 1. General characteristics of study population at baseline** |  |  |
| --- | --- | --- |
| **Variables** | **N/Mean** | **%/SD** |
| **Regional variables** |  |  |
| **Number of thyroid fine-needle aspiration biopsy in Si-Gun-Gu (per 100,000 people)**† | 73.16 | 100.17 |
| **Financial independence rate of local government (%)**† | 65.02 | 25.52 |
| **Individual variables** |  |  |
| **Sex** |  |  |
| Male | 563,671 | 50.22 |
| Female | 558,785 | 49.78 |
| **Age (years)** |  |  |
| 0–19 | 361,444 | 32.20 |
| 20–29 | 167,193 | 14.90 |
| 30–39 | 189,171 | 16.85 |
| 40–49 | 173,275 | 15.44 |
| 50–59 | 100,351 | 8.94 |
| 60–69 | 78,457 | 6.99 |
| 70–79 | 37,855 | 3.37 |
| 80+ | 14,710 | 1.31 |
| **Type of insurance coverage** |  |  |
| Medical Aid | 33,047 | 2.94 |
| NHI (self-employed) | 506,205 | 45.10 |
| NHI (employed) | 583,204 | 51.96 |
| **Income (percentiles)** |  |  |
| 0–29% | 166,071 | 14.80 |
| 30–59% | 273,744 | 24.39 |
| 60%+ | 682,641 | 60.82 |
| **Year of baseline** |  |  |
| 2003 | 1,016,882 | 90.59 |
| 2004 | 21,328 | 1.90 |
| 2005 | 9,945 | 0.89 |
| 2006 | 8,643 | 0.77 |
| 2007 | 10,339 | 0.92 |
| 2008 | 9,669 | 0.86 |
| 2009 | 8,838 | 0.79 |
| 2010 | 9,212 | 0.82 |
| 2011 | 9,474 | 0.84 |
| 2012 | 9,561 | 0.85 |
| 2013 | 8,565 | 0.76 |
| **Region (distance from Seoul)** |  |  |
| Gangwon-do (100.6km) | 34,174 | 3.04 |
| Gyeonggi-do (40.0km) | 241,194 | 21.49 |
| Gyeongsangnam-do (366.4km) | 72,220 | 6.43 |
| Gyeongsangbuk-do (225.5km) | 62,693 | 5.59 |
| Gwangju (295.3km) | 32,940 | 2.93 |
| Daegu (288.3km) | 57,990 | 5.17 |
| Daejeon (160.9km) | 33,746 | 3.01 |
| Busan (394.2km) | 84,435 | 7.52 |
| Seoul | 234,162 | 20.86 |
| Ulsan (395.7km) | 25,572 | 2.28 |
| Incheon (37.7km) | 59,848 | 5.33 |
| Jeollanam-do (346.3km) | 46,425 | 4.14 |
| Jeollabuk-do (216.9km) | 44,658 | 3.98 |
| Jeju-do (541.6km) | 12,857 | 1.15 |
| Chungcheongnam-do (129.9km) | 44,784 | 3.99 |
| Chungcheongbuk-do (137.1km) | 34,758 | 3.10 |
| **Follow-up period (years)** | 9.82 | 2.39 |
| **Total** | 1,122,456 | 100.00 |

† The mean and standard deviation of each continuous variables for study population at baseline
